# Feasibility of abbreviated magnetic resonance imaging (AB-MRI) screening in women with a personal history (PH) of breast cancer

**DOI:** 10.1371/journal.pone.0230347

**Published:** 2020-03-12

**Authors:** Yeong Yi An, Sung Hun Kim, Bong Joo Kang, Young Jin Suh, Ye Won Jeon

**Affiliations:** 1 Department of Radiology, The Catholic University of Korea, St. Vincent’s Hospital, College of Medicine, Suwon-si, Korea; 2 Department of Radiology, The Catholic University of Korea, Seoul St. Mary’s Hospital, College of Medicine, Seoul, Korea; 3 Department of Surgery, The Catholic University of Korea, St. Vincent’s Hospital, College of Medicine, Seoul, Korea; University at Buffalo, UNITED STATES

## Abstract

**Purpose:**

To investigate the feasibility of abbreviated magnetic resonance imaging (AB-MRI) in women with a personal history (PH) of breast cancer as a screening tool.

**Materials and methods:**

We retrospectively reviewed 1880 screening AB-MRIs in 763 women with a PH of breast cancer (median age, 55 years; range, 23–89 years) between October 2015 and October 2016. The total acquisition times of AB-MRI were 8.3 min and 2.8 min with and without T2-weighted imaging, respectively. The tissue diagnosis or one-year follow-up status was used as the reference standard. The characteristics of tumor recurrences detected on AB-MRI screenings were analyzed. The cancer detection rates (CDRs) and additional CDRs for the 1^st^ round and overall rounds of AB-MRI screening were calculated. The recall rate, sensitivity, specificity, positive predictive values for recall (PPV1) and biopsy (PPV3) for the 1^st^ round of AB-MRI screening were calculated. The diagnostic performance of the combination of mammography and ultrasonography was compared with that of AB-MRI by receiver operating characteristic (ROC) curve analysis.

**Results:**

Fifteen of a total of 21 recurrences were detected on the 1^st^ round of AB-MRI screening: 93.3% were node-negative T1 tumors (median tumor size, 1.02 cm; range, 0.1–2 cm) or Tis; 66.7% were high-grade tumors; 8 of these 15 were mammographically and ultrasonographically occult. The CDR and additional CDR for the 1^st^ round of AB-MRI screening were 0.019 and 0.010 per woman, respectively. The sensitivity, specificity, recall rate, PPV1 and PPV3 for the 1^st^ round of AB-MRI screening were 100%, 96.0%, 14.3%, 13.8% and 58.3%, respectively. For detecting secondary cancer, AB-MRI showed a higher sensitivity and PPV than the combination of mammography and ultrasonography (95.2%, 57.1% vs 47.6%, 38.5%). The area under the ROC curve was higher for AB-MRI (0.966; 95% CI: 0.951–0.978) than the combination of mammography and ultrasonography (0.727; 95% CI: 0.694–0.759) (P<0.0001).

**Conclusion:**

AB-MRI improved cancer detection with a high specificity, sensitivity and PPV in women with a PH of breast cancer. AB-MRI could be a useful screening tool for detecting secondary cancer considering its high diagnostic performance and short examination time.

## Introduction

Women with a personal history (PH) of breast cancer are known to be at an increased risk of developing a second breast cancer, which can be local breast cancer recurrence or contralateral breast cancer [1–5]. The purpose of surveillance after primary breast cancer treatment (BCT) is to detect second breast cancer in the early and asymptomatic phase, which is associated with improved patient survival and quality of life [6]. The current guidelines support only mammography (MG) screening for imaging surveillance after BCT [7, 8]. However, the capability of MG for early cancer detection is lower in women with a PH of breast cancer than in women without a PH of breast cancer [9]. Post-treatment changes, such as increased density due to edema or architectural distortion due to scarring or fibrosis, can compromise the ability of MG to detect tumor recurrence early [9, 10]. To overcome the limited sensitivity and higher interval cancer rate of MG in patients after BCT, supplemental imaging examinations, such as ultrasonography (US) or magnetic resonance imaging (MRI), have been increasingly used [11–14]. At this point, there remains much controversy regarding the optimal imaging modality for surveillance in these patient populations.

Breast MRI is well known as the most sensitive imaging modality for detecting breast cancer irrespective of the breast density on MG. Moreover, MRI can detect biologically relevant cancer (invasive cancer or high-grade ductal carcinoma in situ, DCIS) [15]. Despite the advantages of MRI over MG, the widespread use of MRI in breast cancer screening is limited primarily due to its high cost, limited availability, and longer examination and interpretation times. Recently, Kuhl et al. [16] proposed a protocol for abbreviated MRI (AB-MRI) with a short image acquisition time and similar diagnostic accuracy for detecting breast cancer compared to the full diagnostic protocol (FDP) of standard MRI. In subsequent studies, the reported sensitivity and specificity of AB-MRI protocol were comparable to those of the FDP [17–20]. AB-MRI has great potential for cost savings associated with a short scan time and could render MRI competitive with other imaging modalities, such as MG and US, for screening. Recently, the feasibility of AB-MRI for breast cancer screening at different risk levels has been under active investigation. To the best of our knowledge, the role of AB-MRI as a screening tool for surveillance after primary BCT has not been established. Until now, only one study has been reported [21]. In our institution, AB-MRI has been implemented for imaging surveillance after primary BCT since October 2015. We investigated the usefulness of AB-MRI as a surveillance tool in women with a PH of breast cancer and present the outcomes of AB-MRI screening in this population.

## Materials and methods

### Study population

**Fig 1:**
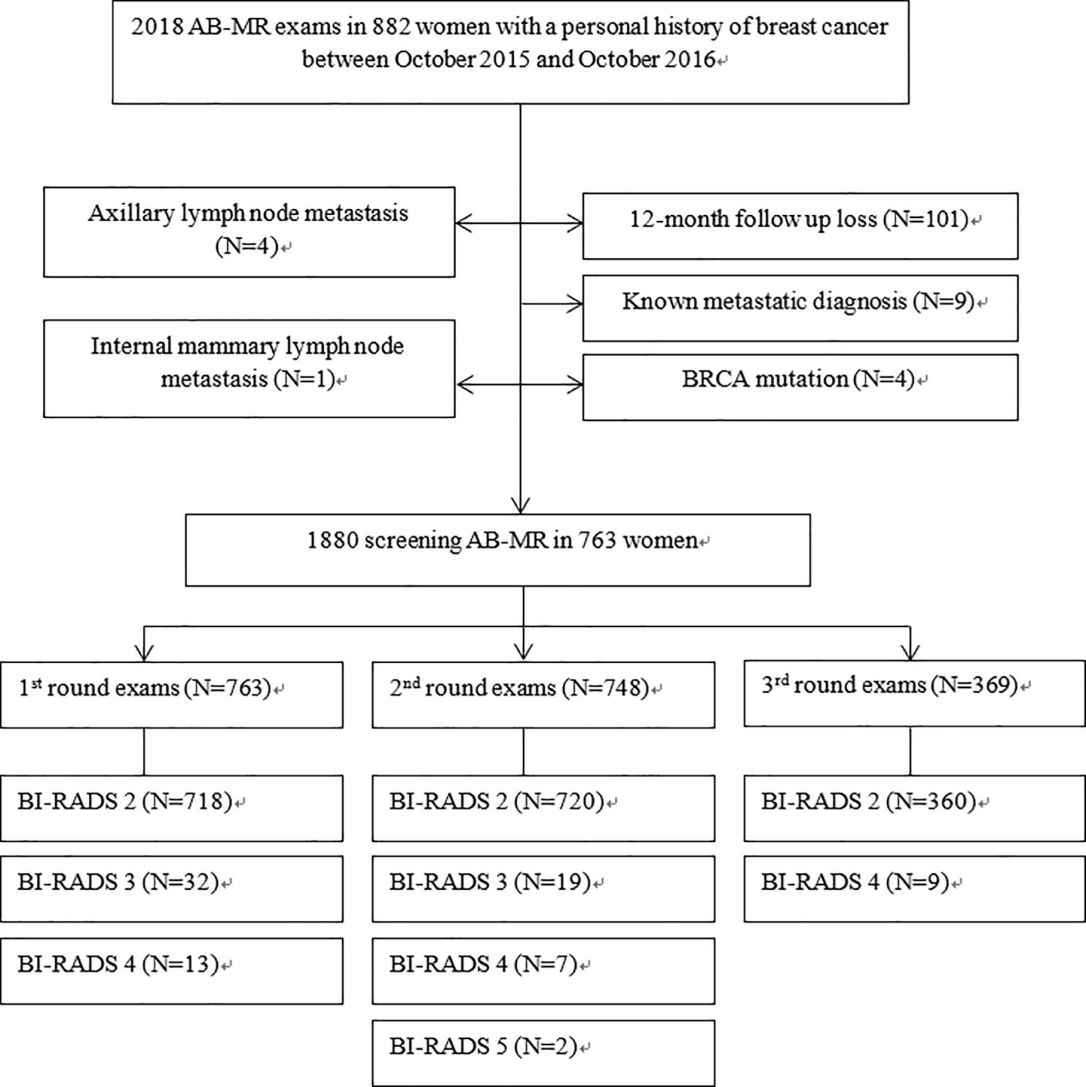
Study population.

This retrospective study was approved by the Catholic Medial Center Office of the Human Research Protection Program (CMC-OHRP)/Institutional Review Board (Approval No. VC17RESI0107), and the requirement for informed consent was waived. Between October 2015 and October 2016, 2018 AB-MRI exams in 882 women with a PH of breast cancer were performed. A total of 119 women were excluded because of BRCA mutation (N = 4), loss to follow-up by 12 months after the 1^st^ round of AB-MRI screening (N = 101), known metastatic disease (N = 9), axillary lymph node metastasis (N = 4) and internal mammary lymph node metastasis (N = 1). A total of 763 women (median age, 55 years; age range, 23–89 years) with 1880 AB-MRI scans were included in our study population **(Fig 1)**. Among the patients, 763 women underwent one round of AB-MRI screening, 748 women underwent two rounds of AB-MRI screening and 360 women underwent three rounds of AB-MRI screening during the study period.

At our institution, following breast cancer surgery, patients underwent follow-up examinations with MG and US every 6 months for the first 2 years and annually thereafter. MRI was not routinely recommended in patients with a PH of breast cancer until AB-MRI was implemented as part of posttreatment surveillance in 2015. AB-MRI examinations were performed along with MG and US examinations on the same day or around the same time. AB-MRI was also performed at the request of patients or clinicians. The median interval between the initial surgery for breast cancer and the first AB-MRI examination was 33.6 months (range, 3–187.5 months). At the time of AB-MRI screening, there was no imaging evidence of malignancy on the previously performed MG and US examinations.

### AB-MRI technique

AB-MRI was performed with the patient in the prone position using a 3 T MR scanner (MAGNETOM Verio, Siemens Medical Solutions, Erlangen, Germany) equipped with a dedicated surface breast coil. The AB-MRI protocols of our institution consisted of axial fat-suppressed, T2-weighted imaging (T2WI), pre- and postcontrast axial T1-weighted imaging (T1WI) before and immediately after gadoterate meglumine injection (at 0.1 mmol per kilogram body weight, Dotarem; Guerbet, Anlnay-Sous-Bois, France), subtraction from postcontrast T1WI and reformatting with a maximum-intensity projection (MIP). The imaging parameters of the 3 T Verio scanner were as follows: (1) turbo spin echo T2WI: TR/TE, 3530/93 ms; slices, 34; FOV, 38 cm; matrix size, 576x403; NEX, 1; slice thickness, 4 mm; and (2) pre- and postcontrast T1WI with a flash 3D VIBE sequence: TR/TE, 3.8/1.4 ms; flip angle, 10°; slice thickness, 1.2 mm with no gap. The total acquisition times were 8.3 min including T2WI and 2.8 min excluding T2WI.

### Image interpretation and outcome analysis

We retrospectively reviewed the clinical history of the patients, AB-MRI findings, preoperative MRI findings for comparison, and findings of other imaging modalities, such as MG and US, when available. Of the 763 women, 50.1% (385 of 768) previously underwent preoperative MRI. The AB-MRI data were interpreted by one of three radiologists with 9–16 years of breast MRI experience using the ACR Breast Imaging Reporting and Data System (BI-RADS) MRI lexicon. BI-RADS 2 included postoperative changes, including seroma or fat necrosis, which were stable over 2 years, cysts, and intramammary lymph nodes and masses assessed as benign by morphology or previous biopsy (focal or oval circumscribed masses with dark internal septation, high signal intensity on T2WI, or a fatty hilum). For probably benign lesions assigned as BI-RADS 3, e.g., a new unique focus with benign morphological features separate from background parenchymal enhancement (BPE) or a mass with benign morphological features, follow-up AB-MRI at 6–12 months was recommended. If lesions were stable during follow-up periods, they were downgraded to the BI-RADS 2 category, but lesions were upgraded to the BI-RADS 4 category if any changes developed. Lesions categorized as BI-RADS 4 or more, e.g., suspicious regions of clumped, linear, or segmental nonmass enhancement or irregular masses with heterogenous or rim enhancement, on AB-MRI were first evaluated by targeted US. If a correlating lesion was present on targeted US, US-guided biopsy was performed. If a mammographic correlation was present, an excisional biopsy after MG-guided needle localization was performed. For lesions occult on both MG and US, MRI-guided biopsy was recommended, but there were no such cases with a lesion only visible on AB-MRI in this study.

The overall and additional cancer detection rate (CDR) for AB-MRI, recall rate, positive predictive value (PPV) for recall (PPV1), PPV for biopsy (PPV3), sensitivity, and specificity of AB-MRI for the 1^st^ round and overall rounds of AB-MRI screening were calculated. The CDR was defined as the number of detected malignancies per 1000 women. A negative AB-MRI examination was defined as BI-RADS 2, and a positive AB-MRI examination was defined as BI-RADS 3, 4 or 5. The reference standard was based on the biopsy or follow-up imaging results within one year after the first round of AB-MRI screening. True positive (TP) was defined as a case with positive AB-MRI findings resulting in a tissue diagnosis of cancer within one year. True negative (TN) was defined as a case with negative AB-MRI findings and the absence of cancer within one year. False positive (FP) was defined as a case with positive AB-MRI findings with no detection of cancer within one year. False negative (FN) was defined as a case with negative AB-MRI findings and a tissue diagnosis of cancer within one year.

The diagnostic performances of AB-MRI screening and the combination of mammography and ultrasonography were evaluated and compared using receiver operating curve (ROC) analyses. Clinicopathologic characteristics of the primary breast cancers were also obtained and compared between women with and without tumor recurrence detected by AB-MRI using the Student t-test or Fisher’s exact test. All computations and statistical analysis were performed using SAS version 9.4 (SAS Institute, Cary, NC, USA) and MedCalc ver. 16.1 (MedCalc software, Mariakerke, Belgium), and P-values <0.05 indicated statistical significance.

## Results

### Characteristics of women with cancer detected by AB-MRI

The demographic details of the study population and the characteristics of the women with cancer detected by AB-MRI are summarized in Table 1. A total of 21 recurrent tumors were diagnosed by AB-MRI screening overall. Women with and without tumor recurrence detected by AB-MRI differed significantly in ER and PR status (Table 1). ER and PR negativity vs positivity was significantly associated with the detection of cancer by AB-MRI (4.9% vs 2%, P = 0.0284 and 4.3% vs 1.4%, P = 0.0150).

**Table 1:** Characteristics of 763 women with a personal history of breast cancer included in this study.

	Women with tumor recurrence detected by AB-MRI (N = 21)	Women without tumor recurrence detected by AB-MRI (N = 742)	
Median age at diagnosis (years)†	53.7 (34–74)	55.0 (23–89)	0.264
<50	8 (3.2)	243 (96.8)	
≥50	13 (2.5)	499 (97.5)	
Op type			0.151
BCS	17 (2.5)	671 (97.5)	
Mastectomy	4 (5.3)	71 (94.7)	
TNM stage			0.191
0 (DCIS)	4 (3.3)	116 (96.7)	
I	10 (2.8)	345 (97.2)	
II	3 (1.3)	221 (98.7)	
III	4 (6.2)	60 (93.7)	
ER			0.0284
(+)	11 (2)	548 (98)	
(-)	10 (4.9)	194 (95.1)	
PR			0.0150
(+)	6 (1.4)	411 (98.6)	
(-)	15 (4.3)	331 (95.7)	
HER-2			0.1734
(+)	9 (4)	216 (96)	
(-)	12 (2.2)	526 (97.8)	
Axillary nodal involvement			0.5467
(+)	4 (2.1)	184 (97.9)	
(-)	17 (3.0)	558 (97.0)	
FGT			0.352
a, b	8 (2.2)	359 (97.8)	
c, d	13 (3.3)	383 (96.7)	
BPE			0.60
Minimal to mild	20 (2.7)	721 (97.3)	
Moderate to marked	1 (4.5)	21 (95.5)	
Interval from surgery to AB-MRI screening (days)	1195.3 (158–3188)	1022 (91–5718)	0.170
<24 months	7 (1.9)	360 (98.1)	
>24 months	14 (3.5)	382 (96.5)	
Preoperative MRI			0.385
(+)	12 (3.1)	373 (96.9)	
(-)	9 (2.4)	369 (97.6)	

*BCS, breast-conserving surgery; DCIS, ductal carcinoma in situ; ER, estrogen receptor; PR, progesterone receptor; HER-2 human epidermal growth factor receptor; BPE, background parenchymal enhancement; AB-MRI, abbreviated magnetic resonance imaging; MRI, magnetic resonance imaging

†Numbers in parentheses are ranges.

### Characteristics of second breast cancer detected on AB-MRI

The type and biological profiles of the detected malignancies are summarized in Table 2. A total of 15 malignancies were diagnosed on the 1^st^ round of AB-MRI screening: 80% (12 of 15) were invasive malignancies (median tumor size, 1.1 cm; range, 0.1–2 cm); 20% (3 of 15) were DCIS (median tumor size, 2.9 cm; range, 1.3–5.5 cm); 93.3% (14 of 15) were Tis or node-negative T1 lesions (median tumor size, 1.02 cm; range, 0.1–2 cm); and 66.7% (10 of 15) were high-grade tumors. On the 2^nd^ round of AB-MRI examinations, 6 malignancies were diagnosed: 83.3% (5 of 6) were invasive malignancies (median tumor size, 0.76 cm; range, 0.3–1.1 cm); 16.7% were DCIS (1 of 6); and 83.3% (5 of 6) were Tis or node-negative T1 lesions. Overall, 17 were invasive malignancies (median tumor size, 1 cm; range, 0.1–2 cm); 4 were DCIS (median tumor size, 2.9 cm; range, 1.3–5.5 cm); 90.5% (19 of 21) were Tis or node-negative T1 lesions; and 57.1% (12 of 21) were high-grade tumors.

**Table 2:** Clinical and imaging characteristics of recurrent tumors detected by AB-MRI in 21 women with a history of breast cancer.

		AB-MRI			Secondary breast cancer						Primary breast cancer		
Pt. No	Age range (years)	Location	Lesion type	BI-RADS category	Size	Histology	Node(+)	Grade	Subtypes	Interval	Histology	TNM stage	Subtype
Recurrent tumor detected on the 1^st^ round of AB-MRI screening (N = 15)													
21	<50	Contralateral	NME	4	2	IDC	+	High	TN	382 (39.2)	IDC	T4N3(IIIC)	TN
41	≥50	Ipsilateral	Mass	4	0.7	IDC	-	High	Lum B	189 (6.2)	IDC	T2N0(IIA)	Lum B
55	≥50	Contralateral	Mass	3	0.6	IDC	-	High	Lum A	2219(72.7)	IDC	T1N0(I)	LumB
58	≥50	Ipsilateral	Mass	3	1	IDC	-	High	HER2(+)	828(27.1)	IDC	T1N0(I)	TN
61	<50	Ipsilateral	Mass	4	1.2	IDC	-	Intermediate	Lum B	1094(35.8)	IDC	T1N0(I)	Lum A
88	<50	Ipsilateral	Mass	5	1.3	IDC	-	High	LumA	427(14)	IDC	T2N2(IIIA)	Lum A
129	<50	Ipsilateral	Mass	3	0.1	IDC	-	High	HER2(+)	158(5.1)	IDC	T1N2(IIIA)	HER2(+)
154	≥50	Ipsilateral	Mass	4	0.5	IDC	-	High	HER2(+)	359(11.7)	IDC	T1N0(I)	Lum B
172	≥50	Ipsilateral	Mass	3	1.6	IDC	-	Low	NA	925(30.3)	ACC	TisN0(0)	TN
261	≥50	Contralateral	NME	4	2	DCIS	-	High	HER2(+)	3053(100)	Mucinous	T2N0(IIA)	LumB
278	<50	Ipsilateral	Mass	3	2	IDC	-	High	TN	180(5.9)	IDC	T1N0(I)	TN
588	<50	Contralateral	NME	3	5.5	DCIS	-	High	HER2(+)	785(25.7)	DCIS	TisN0(0)	HER2(+)
700	≥50	Contralateral	Mass	4	1.3	IDC	-	Low	LumA	1451(47.5)	IDC	T1N0(I)	LumA
710	≥50	Ipsilateral	NME	3	0.9	IDC	-	Intermediate	LumA	1421(46.5)	IDC	T1N0(I)	LumA
715	<50	Contralateral	NME	4	1.3	DCIS	-	Intermediate	HER2(+)	1197(39.2)	DCIS	TisN0(0)	LumA
Recurrent tumor detected on the 2^nd^ round AB-MRI screening (N = 6)													
82	<50	Ipsilateral	NME	3	3	DCIS	-	High	HER2(+)	159(5.2)	IDC	T1N0(I)	TN
152	≥50	Ipsilateral	NME	3	0.3	IDC	-	Intermediate	NA	940(30.8)	IDC	T1N0(I)	HER2(+)
158	≥50	Contralateral	NME	3	1.1	IDC	+	Intermediate	HER2(+)	1790(58.6)	DCIS	TisN0(0)	LumB
164	≥50	Ipsilateral	Mass	4	0.5	IDC	-	Intermediate	HER2(+)	1086(35.6)	DCIS	TisN0(0)	HER2(+)
314	≥50	Contralateral	Mass	3	1.1	IDC	-	High	LumA	1278(41.9)	IDC	T1N0(I)	LumA
614	≥50	Contralateral	Mass	4	0.8	IDC	-	Intermediate	TN	2919(95.7)	IDC	T2N3(IIIC)	TN

*IDC, invasive ductal carcinoma; DCIS, ductal carcinoma in situ; ACC, adenoid cystic carcinoma; TN, triple negative; Lum A, luminal A; Lum B, luminal B

### Cancer detection yield

Fifteen malignancies were detected on the 1^st^ round of AB-MRI screening, and the remaining 6 malignancies were detected on the 2^nd^ round of AB-MRI screening. Among the 763 1^st^ round of AB-MRI screening examinations, the final BI-RADS categories were as follows: BI-RADS 2 in 654 examinations (85.7%), BI-RADS 3 in 96 examinations (12.6%), and BI-RADS 4 in 13 examinations (1.7%). Of the 13 BI-RADS 4 lesions, 12 showed correlating lesions on US and were biopsied under US guidance; 7 malignancies and 5 benign lesions were diagnosed. One patient with a nonmass lesion on AB-MRI denied biopsy, but this lesion showed regression on follow-up examinations. Of the 96 BI-RADS 3 lesions, 11 were biopsied under US guidance either because they showed progression on follow-up examinations (n = 7) or the patient requested biopsy (n = 4); 8 malignancies and 3 benign pathologies were diagnosed. Eight of 15 malignancies diagnosed on the 1^st^ round of AB-MRI screening were occult on MG and US. Thus, the CDR of the 1^st^ round of AB-MRI screening was 0.019 per woman (15 of 763, 7+8/763), and the additional CDR of the 1^st^ round of AB-MRI screening was 0.010 per woman (8 of 763). The recall rate was 14.3% (96+13/763). The PPV1 was 13.8% (15 of 109; BI-RADS 3+4 = 96+13), and the PPV3 was 58.3% (7 of 12). There were no FN cases. The sensitivity, specificity, and accuracy of the 1^st^ round of AB-MRI screening was 100%, 95.99% and 96.07%, respectively.

Seven lesions were newly detected on 2^nd^ round AB-MRI screening, and 6 malignancies were diagnosed. Two BI-RADS 4 lesions underwent US-guided biopsy because they showed a correlating lesion on US; one was a malignancy, and one was benign. The remaining 5 BI-RADS 3 lesions were upgraded to BI-RADS 4 on the next follow-up AB-MRI examination and underwent US-guided biopsy because they showed a correlating lesion on US; all 5 lesions were diagnosed as malignancies. Two lesions were newly detected on 3^rd^ round AB-MRI screening, but all were diagnosed as benign pathologies.

A total of 32 lesions were biopsied; 21 malignancies and 11 benign pathologies were diagnosed based on 1880 AB-MRI screening examinations in 763 patients. Eleven of 21 malignancies were only visible on AB-MRI. Thus, the overall CDR of AB-MRI including all screening rounds was 0.027 per woman (21/763), and the additional CDR was 0.014 per woman (11/763).

### Comparison of diagnostic performance between MRI and the combination of MG and US

**Fig 2:**
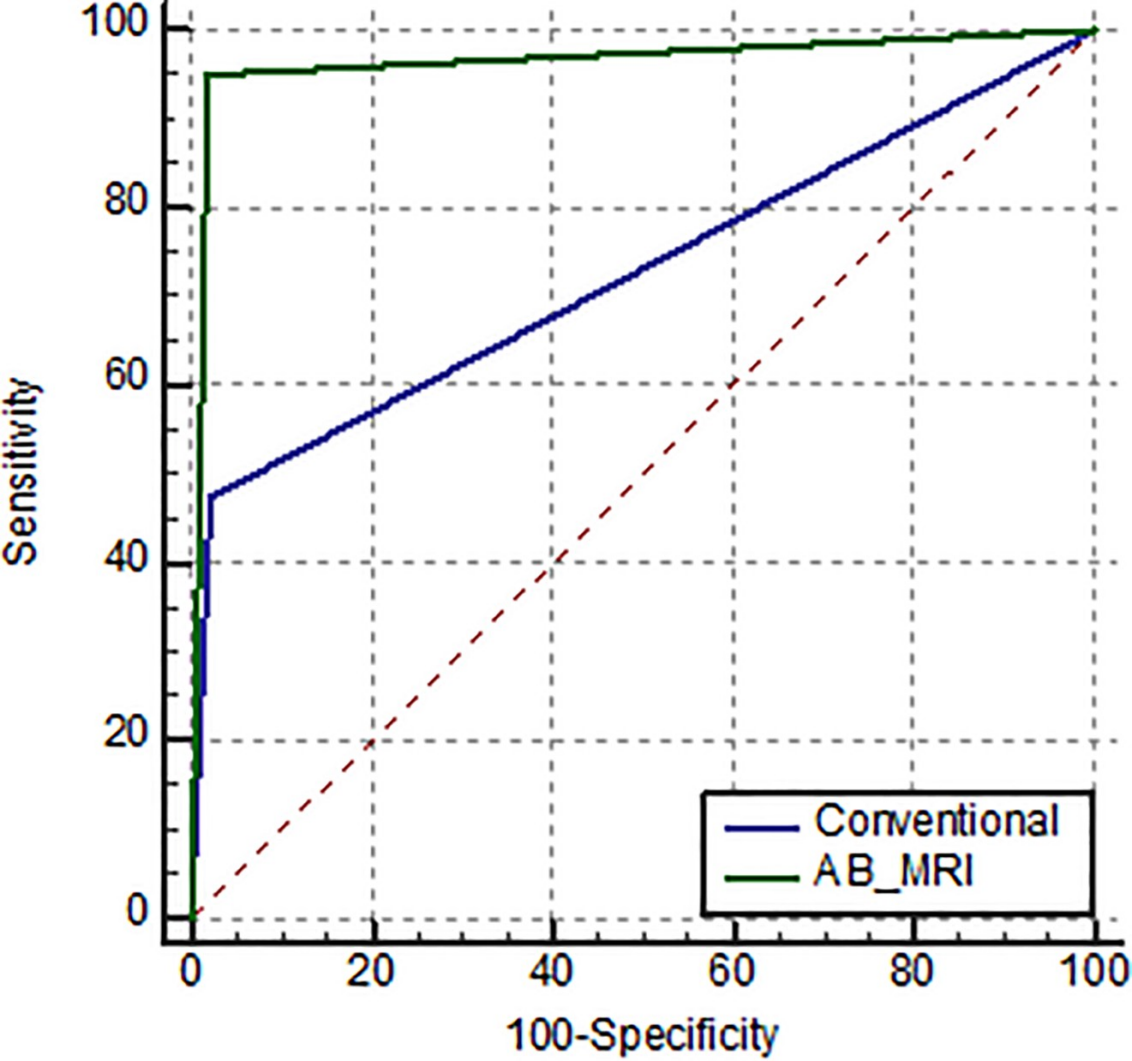
Comparison receiver operating characteristic curve analysis (P<0.0001).

A comparison of the diagnostic performance between conventional imaging and AB-MRI is shown in Table 3, and the corresponding ROC curve analysis is shown in Fig 2. AB-MRI showed a higher sensitivity and PPV than the conventional combination of MG and US (95.2%, 57.1% vs 47.6%, 38.5%). The specificity, NPV and accuracy were similar for AB-MRI and the combination of MG and US. ROC curve analysis showed that the area under the ROC curve (AUC) was significantly higher for AB-MRI (0.966; 95% CI; 0.951–0.978) than the combination of MG and US (0.727, 95% CI; 0.694–0.759) (P<0.0001).

**Table 3:** Comparison of diagnostic performances between AB-MRI and the combination of MG and US for detecting tumor recurrence (overall screening rounds).

Modality	Sensitivity	Specificity	PPV	NPV	AUC	SE	Accuracy
Conventional imaging (MG+US)	47.6%	97.8%	38.5%	98.5%	0.727 (0.694, 0.759)	0.0559	96.5%
AB-MRI	95.2%	98.0%	57.1%	99.9%	0.966 (0.951, 0.978)	0.239	97.9%

* PPV, positive predictive value; NPV, negative predictive value; AUC, area under ROC curve; SE, standard error

## Discussion

The ACS and NCCN guidelines recommend breast MRI screening for women in high-risk groups, including those who with more than a 20–25% lifetime risk of developing breast cancer, who are carriers or have first-degree relatives with a BRCA mutation, or who have had chest radiation therapy [22–24]. Although women with a PH of breast cancer have a substantially increased risk of developing second breast cancer [5, 25–27], the current guidelines do not include recommendations either for or against MRI screening in this intermediate-risk group due to insufficient performance data and concerns of costs and unnecessary recall and biopsy rates [22–24]. However, recent studies have suggested that a PH is a risk factor similar to a genetic or family history and that women with a PH can also benefit from MRI screening with increased CDRs [13, 28, 29]. The reported sensitivity, specificity and PPV of MRI screening in women with a PH were 88.5%, 94% and 12.3%, respectively [28, 29], which are equivalent to those in high-risk patients. Considering the limited sensitivity of MG and the harm of FN findings after BCT, growing interest in the utility of high-sensitivity breast MRI is not surprising in women with a PH of breast cancer.

Several previous investigations have demonstrated relatively consistent results, although the range in the reported data is somewhat broad and heterogeneous due to differences in patient selection, study design and applied MRI protocols (Table 4) [13, 21, 28–38]. MRI showed higher incremental cancer yields (CDR, ranging from 3.8 to 118.1 per 1000 women) [13, 21, 28–38], a high sensitivity of 75–100%, a high specificity of 82.2–98.3% [21, 28, 29, 31–38], a PPV1 of 5.3–20.3% and a PPV3 of 15.8–61.5% [21, 28,29, 31–38]. Most studies used the standard breast MRI FDP, but its long exam length is likely one of the major barriers to the more widespread utilization of breast MRI for screening purposes. The first investigation that evaluated the utility of the short AB-MRI protocol in women with a PH of breast cancer was a recent study by Choi et al [21], which retrospectively analyzed 799 AB-MRI exams in 725 women. Compared with their study, our study shows a similar recall rates and PPVs, slightly higher CDRs and a higher specificity with similar examination times. The results of both studies support the finding that AB-MRI offers substantially increased CDRs with high sensitivity and without sacrificing specificity. The recall rates and PPVs of AB-MRI are within the range considered acceptable for MG screening [16, 39,40]. Until now, the study by Cho et al. was the only prospective trial in a cohort of women with a PH of breast cancer under 50 years of age at initial diagnosis who underwent dynamic contrast-enhanced (DCE)-MRI [34]. In this study, the CDR of MRI was 8.8 per 1000 screens, which is clearly below the range reported in previous retrospective studies, including ours [13, 21, 28–33, 35, 37,38]. However, CDR overestimation in single-center retrospective studies might be due to selection bias. Although both studies by Choi et al [21] and our own study show the usefulness of AB-MRI in women with a PH of breast cancer, further prospective, randomized, multicenter studies are required to validate its applicability.

**Table 4:** Studies of MRI screening in women with a PH of breast cancer.

Studies	Year	No. of patients	MRI protocol	CDR	Recall rate (%)	Sensitivity (%)	Specificity (%)	PPV1 (%)	PPV3 (%)
Brennan et al. [30]	2010	144	FDP	118.1^b^	NA	NA	NA	NA	38.6
Elmore et al. [35]	2010	141^a^	FDP	14.1^b^	11.3	100	89.9	NA	NA
Schacht et al. [13]	2014	208	FDP	28.8^b^	NA	NA	NA	NA	NA
Gweon et al. [32]	2014	607	FDP	18.1^b^^,^^c^	19.3 ^b^^,^^c^	91.7	82.2	9.4	43.5
Giess et al. [31]	2015	691^a^	FDP	10.1^d^	10.7	100	89.9	9.4	17.9
Weinstock et al. [33]	2015	249	FDP	19.3^b^	NA	84.6	95.3	4.4	25.6
Lehman et al. [28]	2016	915	FDP	19.7^b^	14.3	80	94	14.3	25
Destounis et al. [29]	2016	131	FDP	39.4 ^d^	19.4	100	83.9	20.3	28.8
Cho et al. [34]	2017	754	FDP	7.3^d^	10.7	88.2	89.9	6.8	23.5
Tadros et al. [38]	2017	186	EDP	43 ^b^	NA	100	94.6	NA	23.5
Choi et al. [21]	2018	725	AB	15^d^	12.1	100	89.2	12.4	61.5
Park et al. [36]	2018	1044	FDP	3.8^d^	7.2	75	98.3	5.3	15.8
Vreemann et al. [39]	2018	836	FDP	13.6^d^	47.2	82.2	96.5	26	36
An et al.	2019	763	AB	19^,^^c^	14.3	100	96.0	13.8	58.3

^a^Studies including women with additional risk (family history of gene mutation)

^b^Calculated per 1000 women

^c^Calculated for the first screening round

^d^Calculated per 1000 exams

*MRI, magnetic resonance imaging; CDR, cancer detection rate; PPV1, positive predictive value for recall; PPV3, positive predictive value for biopsy; FDP, full diagnostic protocol; AB, abbreviated MRI protocol; NA, not applicable

In our study, 93.3% of detected malignancies were node-negative T1 lesions (median tumor size, 1.02 cm; range, 0.1–2) or Tis on the 1^st^ round of AB-MRI screening, and 66.7% were high-grade tumors. Overall, i.e., including the 1^st^ and 2^nd^ round of AB-MRI screening, 90.5% were node-negative T1 malignancies (median tumor size, 0.93 cm; range, 0.1–2) or Tis, and 61.9% were high-grade tumors. The outcomes of our study show good agreement with the results of previous studies of high-risk women [11, 15] and women with a PH of breast cancer [21, 30, 32] in that most lesions were node negative, and the median size of invasive tumors was 0.7–1.8 cm. In addition, we found that both ER and PR negativity was significantly associated with cancer detected on AB-MRI, which is similar to the result reported by Gweon et al [32]. Thus, our findings also indicate that malignancies detected on MRI are usually early stage, node negative and biologically significant in women with a PH of breast cancer.

We found that an additional advantage of AB-MRI screening was the ability to detect extramammary abnormalities. In our study, one patient had level III axillary node metastasis, which was located in the infraclavicular area medial to pectoralis minor. Another patient had internal mammary lymph node metastasis. Although those cases were excluded from the analysis, extramammary lesions not covered by MG screening could be detected on AB-MRI, which is an additional advantage of AB-MRI screening.

This study has several limitations. First, this was a retrospective study from a single institution. Selection bias could have affected the true cancer yield of AB-MRI, which might limit the generalizability of our results. Second, our institution recently implemented AB-MRI screening into surveillance protocols. Therefore, the interval between the initial surgery and the 1^st^ round of AB-MRI screening varied. Third, we did not evaluate the effect of using T2WI for the evaluation of incidental breast lesions in decreasing unnecessary recall and excluding FP findings. Fourth, we could not evaluate the appropriate interval/frequency, the cost effectiveness or the survival benefit of AB-MRI screening. Continued prospective, randomized, multicenter research is needed for the wide application of AB-MRI screening in this population.

In conclusion, our data suggest that AB-MRI can improve cancer detection with a short image acquisition time and high diagnostic performance in women with a PH of breast cancer. Furthermore, AB-MRI can depict biologically relevant cancer at an early stage in women with a PH of breast cancer. AB-MRI can be considered a useful postoperative surveillance tool in women with a PH of breast cancer.

## Supporting information

S1 Dataset The basic dataset of this study. This is the basic dataset of this study including comparison ROC analysis.
(XLSX)
